# Care of the Expectant Mother

**Published:** 1903-09

**Authors:** 


Care of the Expectant Mother.—A Treatise on the Care of the Mother During Pregnancy and Child-birth, and Care of the Child From Birth Until Puberty. By W. Lewis Howe, M. D. Sixty-three pages. Published by F. A. Davis Company, Philadelphia.

This short and practical little volume is intended to be given out by the physician to his pregnant patients for their guidance. It endeavors to educate them to the hygienic care of that condition, in so far as they can safely manage it, and to teach them when a physician should be called. The medical terms used throughout the book are, unfortunately, most too technical for the average house-wife and the author several times calls upon a diagnostic ability in her which she could hardly be expected to possess; but on the whole the little volume is well gotten up, and it would be decidedly to every doctor's advantage to have several of these little books for distribution. J. M. L.

